# Influence of Aging on the Retina and Visual Motion Processing for Optokinetic Responses in Mice

**DOI:** 10.3389/fnins.2020.586013

**Published:** 2020-12-01

**Authors:** Yuko Sugita, Haruka Yamamoto, Yamato Maeda, Takahisa Furukawa

**Affiliations:** Laboratory for Molecular and Developmental Biology, Institute for Protein Research, Osaka University, Osaka, Japan

**Keywords:** aging, retina, eye movement, optokinetic response, electroretinogram, visual function, photoreceptor cells, horizontal cells

## Abstract

The decline in visual function due to normal aging impacts various aspects of our daily lives. Previous reports suggest that the aging retina exhibits mislocalization of photoreceptor terminals and reduced amplitudes of scotopic and photopic electroretinogram (ERG) responses in mice. These abnormalities are thought to contribute to age-related visual impairment; however, the extent to which visual function is impaired by aging at the organismal level is unclear. In the present study, we focus on the age-related changes of the optokinetic responses (OKRs) in visual processing. Moreover, we investigated the initial and late phases of the OKRs in young adult (2–3 months old) and aging mice (21–24 months old). The initial phase was evaluated by measuring the open-loop eye velocity of OKRs using sinusoidal grating patterns of various spatial frequencies (SFs) and moving at various temporal frequencies (TFs) for 0.5 s. The aging mice exhibited initial OKRs with a spatiotemporal frequency tuning that was slightly different from those in young adult mice. The late-phase OKRs were investigated by measuring the slow-phase velocity of the optokinetic nystagmus evoked by sinusoidal gratings of various spatiotemporal frequencies moving for 30 s. We found that optimal SF and TF in the normal aging mice are both reduced compared with those in young adult mice. In addition, we measured the OKRs of *4.1G-*null (*4.1G*^–/–^) mice, in which mislocalization of photoreceptor terminals is observed even at the young adult stage. We found that the late phase OKR was significantly impaired in *4.1G*^–^*^/^*^–^ mice, which exhibit significantly reduced SF and TF compared with control mice. These OKR abnormalities observed in *4.1G*^–^*^/^*^–^ mice resemble the abnormalities found in normal aging mice. This finding suggests that these mice can be useful mouse models for studying the aging of the retinal tissue and declining visual function. Taken together, the current study demonstrates that normal aging deteriorates to visual motion processing for both the initial and late phases of OKRs. Moreover, it implies that the abnormalities of the visual function in the normal aging mice are at least partly due to mislocalization of photoreceptor synapses.

## Introduction

Visual function impairments that accompany aging are commonly observed, causing difficulties in the daily lives of elderly people. Structural changes and decreases in electroretinogram (ERG) responses in the retina due to aging have been reported (Gresh et al., 2003; Liets et al., 2006; Williams and Jacobs, 2007; Kolesnikov et al., 2010). In the aging retina, mislocalization of synapses, where the photoreceptor cell terminus makes contact with the dendritic terminus of bipolar and horizontal cells, was observed in both mice and humans (Liets et al., 2006; Eliasieh et al., 2007; Samuel et al., 2011). ERG analysis revealed that the amplitudes of rod- and cone-mediated responses are reduced in aging mice and humans (Gresh et al., 2003; Williams and Jacobs, 2007; Kolesnikov et al., 2010). These reports suggested that age-related changes were observed in both rod and cone photoreceptor systems.

Optokinetic responses (OKRs) are reflexive eye movements induced by a moving visual pattern (Collewijn, 1991; Distler and Hoffmann, 2003; Stahl, 2004, 2008; Leigh and Zee, 2006; Buttner and Kremmyda, 2007; Kodama and du Lac, 2016). When a visual pattern starts to move, the eyes begin to move toward the direction of the stimulus motion to produce an initial phase OKR. If the movement of the visual pattern continues for a longer duration, slow tracking eye movements are interrupted by prompt eye movements that reset the eye to its primary position, thus constituting a late-phase OKR. This ocular response of alternating slow tracking and quick resetting eye movements is also known as optokinetic nystagmus (OKN).

Neural circuits involved in the generation of OKR differ between primates (foveate) and afoveate animals, including mice (Collewijn, 1991). While cortical structures are known to be involved in the generation of OKR in primates (Fuchs and Mustari, 1993), OKR in afoveate animals are considered to be controlled by subcortical structures (Kato et al., 1988; Hoffmann and Distler, 1989; Mustari and Fuchs, 1989, 1990). The cortical structures include the striatum and extrastriatum middle temporal (MT) area/medial superior temporal (MST) area in monkeys and the MT region in humans (Dürsteler and Wurtz, 1988; Bucher et al., 1997; Galati et al., 1999; Kansaku et al., 2001; Takemura et al., 2007). The subcortical structure includes the pretectum (nucleus of the optic tract; NOT) and the accessory optics (AOS) (Kato et al., 1988; Hoffmann and Distler, 1989; Mustari and Fuchs, 1989, 1990). In afoveate mammals, including rabbits and rats, OKRs are unaffected by lesions in the cerebral cortex, suggesting that OKRs are dominated by subcortical structures (Hobbelen and Collewijn, 1971; Harvey et al., 1997). However, the subcortical mechanisms underlying the generation of OKR appear to be common between foveate and afoveate mammals (Wallman, 1993). In rhesus monkeys with occipital lobectomies, the OKR was significantly altered but not abolished, and the remaining OKR was from the foveal animal with a predominant temporal to nasal (T–N) direction during monocular viewing, suggesting that the subcortical structure is also involved OKRs in foveate animals (Zee et al., 1987).

The mouse, an afoveate animal, has long been used to study the effects of retinal abnormalities on OKRs (Yoshida et al., 2001; Cahill and Nathans, 2008; Sato et al., 2008). In the retina, motion direction is detected by direction-selective ganglion cells (DSGCs), that provide direct inputs to the NOT and AOS (Oyster et al., 1980; Simpson, 1984; Dann and Buhl, 1987; Pak et al., 1987; Wallman, 1993; Yonehara et al., 2009; Kim et al., 2010; Kay et al., 2011). Among several types of DSGCs identified (Barlow et al., 1964; Levick, 1967; Wei and Feller, 2011; Vaney et al., 2012), ON and ON-OFF DSGCs are thought to be closely associated with OKRs (Weng et al., 2005; Sun et al., 2006; Vaney et al., 2012). The initial phase of OKRs depends on both ON and ON-OFF DSGCs, whereas the late phase of OKRs relies solely on ON DSGCs in the ON pathway (Sugita et al., 2013).

A membrane scaffold protein, 4.1G, is highly expressed in retinal photoreceptor cells and is required for the formation of photoreceptor synapses at the correct locations (Sanuki et al., 2015). The retinas of *4.1G*^–^*^/^*^–^ mice exhibit mislocalization of photoreceptor synaptic terminals, while their synaptic connections are normally formed. *4.1G*^–^*^/^*^–^ mice exhibit impairments in OKN, demonstrating the importance of correct synaptic location in visual acuity. The aging mouse and human retinas exhibit ectopic synapses (Eliasieh et al., 2007; Samuel et al., 2011) and impaired visual function (Wong and Brown, 2007; van Alphen et al., 2009), which are similarly observed in *4.1G*^–^*^/^*^–^ mice. These results suggest that aging causes abnormalities of the ON pathway in late-phase OKRs. Since previous studies, which examined the effects of aging on OKRs, measured only late-phase OKRs, the detailed visual characteristics throughout the initial and late phases of OKRs affected by aging remain unclear.

To understand aging-induced changes in retinal visual motion processing, both the initial and late phases of OKRs in the aging mice were caused by moving sinusoidal grating patterns at a wide range of spatiotemporal frequencies. In addition, we measured detailed OKRs in *4.1G*^–/–^ mice. Our results indicate that optimal spatiotemporal frequencies are reduced in aging mice compared with those in young adult mice.

## Materials and Methods

### Animal Preparation

All recombinant mouse experimental procedures were approved by the Animal Experimental Committees of the Institute for Protein Research (approval ID 29-01-3) and the Institutional Safety Committee on Recombinant DNA Experiments (approval ID 4220) of Osaka University. The procedures were performed in compliance with institutional guidelines. Mice were housed in a temperature-controlled room at 20–26°C with a 12-h light/dark cycle. Fresh water and rodent diet were available at all times. The young adult mice used in this study were C57BL/6J male mice which were 2–3 months old (18.3–22.2 g body weight), and the aging mice used in this study were C57BL/6J male mice which were 21–24 months old (33.8–40.0 g body weight). *4.1G*^–/–^ mice were bred in the genetic background 129Sv/Ev (Sanuki et al., 2015). Data were obtained from *4.1G*^–/–^ mice and wild-type 129Sv/Ev (Taconic) mice which were 2–3 months old (19.5–25.5 g body weight).

### Immunohistochemistry

Immunohistochemical analysis was conducted, as described previously (Yamamoto et al., 2020). Mouse eyecups were fixed with 4% paraformaldehyde in phosphate-buffered saline (PBS) for 30 min at room temperature. The retinas were cryoprotected by immersing in 30% sucrose/PBS, embedded in an OCT compound (Sakura), and frozen. Then the retinas were sectioned at 20 μm. The tissue sections were treated with PBS, incubated in a blocking solution (4% normal donkey serum and 0.05% Triton X-100 in PBS) for 1 h at room temperature, and then labeled with the primary antibodies in a blocking solution at 4°C overnight. The sections were then washed with PBS and incubated with secondary antibodies in a blocking solution for 2 h at room temperature. We observed the tissue sections under a confocal laser microscope (LSM700; Carl Zeiss). The following antibodies were used for immunostaining: mouse monoclonal anti-PSD95 (1:200, Thermo Fisher Scientific, #MA1-046); anti-Ctbp2 (1:500, BD Biosciences, #612044); rabbit polyclonal anti-Calbindin (1:500, Millipore, #PC253L); and anti-PKCα (1:500, Sigma-Aldrich, #P4334) antibodies. Cy3-conjugated secondary antibodies (1:500, Jackson, #715-165-150) or Alexa Fluor 488-conjugated secondary antibodies (1:500, Invitrogen, #A21206) were used.

### ERG Recording

Electroretinograms were conducted, as described previously (Chaya et al., 2019; Kozuka et al., 2019). In brief, mice were dark-adapted overnight and then we anesthetized the mice by intraperitoneally injecting 100 mg/kg ketamine and 10 mg/kg xylazine. We measured ERG responses using the PuREC system with LED LS-100 (Mayo Corporation). We dilated pupils using topical 0.5% tropicamide and 0.5% phenylephrine HCl. We used four levels of stimulus intensities ranging from −4.0 to 1.0 log cd s/m^2^ to measure the scotopic ERGs. After we light-adapted mice for 10 min, we recorded the photopic ERGs on a rod-suppressing white background of 1.3 log cd s/m^2^. We used four levels of stimulus intensities ranging from −0.5 to 1.0 log cd s/m^2^ to measure the photopic ERG recordings. We averaged eight responses at −4.0 log cd s/m^2^ and four responses at −3.0 log cd s/m^2^ for scotopic recordings. We averaged total 16 responses for photopic recordings.

### Recording of Eye Movement

We illuminated the mouse right eye using infrared light-emitting diodes and monitored using a CCD camera (lens: VS-MC0510, VS Technology, Tokyo, Japan: controller: HR50, Sony, Tokyo, Japan; Figure 4A). The data obtained from the eye movements were analyzed with a computer (Endeavor, Epson, Nagano, Japan) carrying an image-processing software (Geteye, Matsuura-Denko-sha, Kanazawa, Japan). This software identified pupil center and measured its position at intervals of 5 ms (Tabata et al., 2010). We recorded in darkness to avoid contamination from irrelevant visually driven eye movements.

### Visual Stimulation

Visual stimuli were displayed on 19-inch computer screens (spatial resolution, 1280 × 1024 pixels; refresh rate, 75 Hz; LCD, Mitsubishi, Tokyo, Japan). We set three displays showing the identical visual stimulus around the mouse at the front and both sides, spanning 270° × 76.6° (azimuth × height) in the visual field. Each display was set at a distance of 19 cm from the center of the platform on which the head of the animal was stereotaxically affixed. The mouse eyes were positioned 13 cm above the platform. We used drifting vertical sinusoidal gratings (Michelson contrast, 64%; mean luminance, 100 cd/m^2^) for the visual stimuli generated using MATLAB (MathWorks, Natick, MA, United States) and the Psychophysics Toolbox extensions (Brainard, 1997). The SF of the grating on the computer displays was adjusted to mimic a rotating drum drifting at a constant speed, as described previously (Tabata et al., 2010; Sugita et al., 2012, 2013, 2015, 2020; Watanabe et al., 2015; Chaya et al., 2017; Ueno et al., 2018).

### OKR Procedures

We investigated the initial and late phases of OKRs. We used vertical sinusoidal gratings that could have one of five SFs selected randomly from a lookup table: 0.031, 0.063, 0.125, 0.25, and 0.5 cycles/deg in a given trial for the visual images. The visual stimulus speed was defined by TF, which was chosen from 0.1875, 0.375, 0.75, 1.5, 3, 6, 12, or 24 Hz (Figure 4B). For the initial phase of the OKRs, the duration of the visual stimulus motion was 500 ms. At least nine and an average of 18 trials were repeated for each stimulus condition and each mouse. Data were collected from 20 mice [young adult mice (*n* = 5), aging mice (*n* = 5), wild-type mice (*n* = 5), and *4.1G*^–/–^ mice (*n* = 5)].

To investigate the late-phase of the OKRs (OKNs), we analyzed the OKRs eclicited by longer visual motion stimulations (30 s). The image speeds and patterns were exactly the same as those used in the initial phase OKRs. Trials were repeated one to two times for each stimulus condition, and data was obtained from 20 mice [young adult mice (*n* = 5), aging mice (*n* = 5), wild-type mice (*n* = 5), and *4.1G*^–/–^ mice (*n* = 5)].

At the beginning of each trial in the initial and late phases of OKRs, a vertical sinusoidal grating pattern was exhibited on the monitors. The grating pattern was maintained for 333 ms and then started to move either nasal-temporal (N-T) direction or temporal-nasal direction (T-N) for the right eye (Sugita et al., 2013). The visual stimulus speed was fixed during each trial (Figure 4C).

### Data Analysis

All data regarding OKR recordings were analyzed with computer programs which are based on MATLAB (The MathWorks, Inc.). We smoothed the eye-position data using a four-pole digital Butterworth filter (−3 dB at 15 Hz), and then the eye velocity traces were extracted from the two-point backward difference. Eye acceleration profiles were also extracted from the two-point backward difference of the eye velocity traces.

To analyze the initial phase OKRs, we calculated the mean eye velocity during the 100-ms interval starting 100 ms following the onset of visual motion. We discarded trials with saccadic intrusions (eye velocity >30 deg/s, eye acceleration >2000 deg/s^2^) during the 300-ms interval starting 100 ms before the onset of the visual motion. To elevate the signal-to-noise (S/N) ratio, we subtracted the mean eye velocity for N-T motion from the mean eye velocity for T-N motion of the same visual stimulus for each mouse. Since T-N eye movements were positive in our sign convention, the (T-N) – (N-T) eye velocities were positive when the OKR was in the direction of the visual motion. Note that binocular stimulations were used and systematic directional asymmetry was not considered. We averaged data over mice of the same class for each stimulus condition (Sugita et al., 2013). We characterized the spatiotemporal frequency tuning of the responses using two-dimensional (2D) Gaussian functions of log-spatiotemporal frequency:

(1) $$R\,\left(x,y\right)=r\cdot e\,x\,p\,\left(-\left(\sigma_{x}^{2}\,{\left(x-x_{0}\right)}^{2}+2\,\rho \,\sigma_{x}\,\sigma_{y}\,\left(x-x_{0}\right)\,\left(y-y_{0}\right)+\sigma_{y}^{2}\,{\left(y-y_{0}\right)}^{2}\right)\right)$$

where *x*_0_ and *y*_0_ denote the peak SF and TF, respectively, and we optimized along with all the other free parameters, i.e., *r*, *σ_*x*_*, *σ_*y*_*, and *ρ*.

To evaluate the late-phase of the OKRs, we analyzed the optokinetic nystagmus and the slow-phase eye velocity of the OKN. The extraction of slow phases was performed, as described previously (Sugita et al., 2013, 2015; Watanabe et al., 2015). We averaged the slow-phase eye velocity during the 20-s intervals starting 10 s after the onset of the visual motion for each stimulus condition. When more than two trials were available for a stimulus condition, we calculated the average of these trials. We then evaluated the spatiotemporal tuning following the same procedure as was used in the initial phase OKRs.

## Results

### Ectopic Processes Were Observed in the Normal Aging Mouse Retina

**FIGURE 1:**
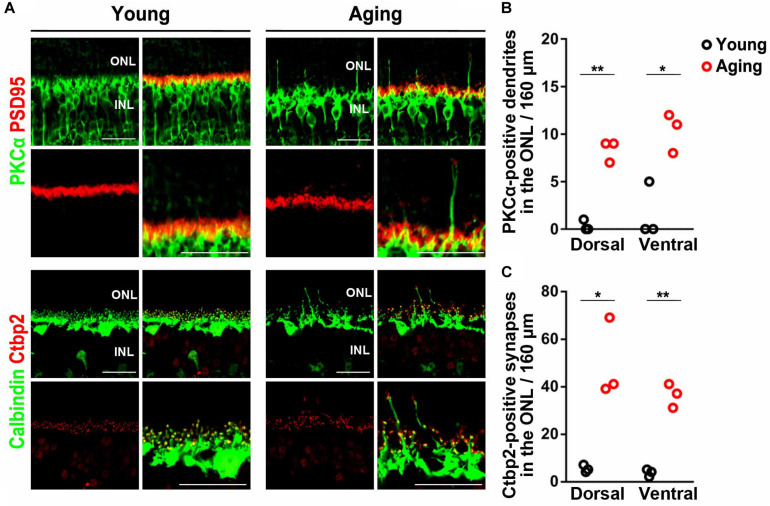
Mislocalization of photoreceptor synaptic terminals observed in aging mouse retinas. **(A)** Retinal sections of the young adult and aging mice were immunostained using anti-PSD95, anti-Ctbp2, anti-Calbindin, and anti-PKCα antibodies. **(B–C)** Extended rod bipolar cell dendrites and ectopic photoreceptor terminals were counted. Student *t*-test (*p < 0.05, **p < 0.01). Error bars, mean ± SD (*n* = 3 mice). ONL, outer nuclear layer; OPL, outer plexiform layer; INL, inner nuclear layer. Scale bars, 25 μm.

Rod bipolar cell dendrites and horizontal cell processes have been observed to extend beyond the outer plexiform layer (OPL) into the outer nuclear layer (ONL) in both the aging mice and humans (Liets et al., 2006; Eliasieh et al., 2007; Terzibasi et al., 2009). To examine the morphology of photoreceptor cell synapses in aging mice, we immunostained young adult and aging adult retinas using antibodies against rod bipolar cell (PKCα), horizontal cell (calbindin), and photoreceptor synapse (PSD95 and Ctbp2) markers (Figure 1A). Consistent with previous reports (Liets et al., 2006; Eliasieh et al., 2007; Terzibasi et al., 2009), we observed a significant increase in rod bipolar cell dendrites extending beyond the OPL into the ONL in the aging retina (Figures 1A,B). Horizontal cell processes also extended into the ONL in the aging retina. Moreover, ectopic photoreceptor terminals in the dorsal and ventral ONL were significantly more observed in the aging retina than in the young adult retina (Figures 1A,C).

### Light Responses Decreased in the Normal Aging Mice

**FIGURE 2:**
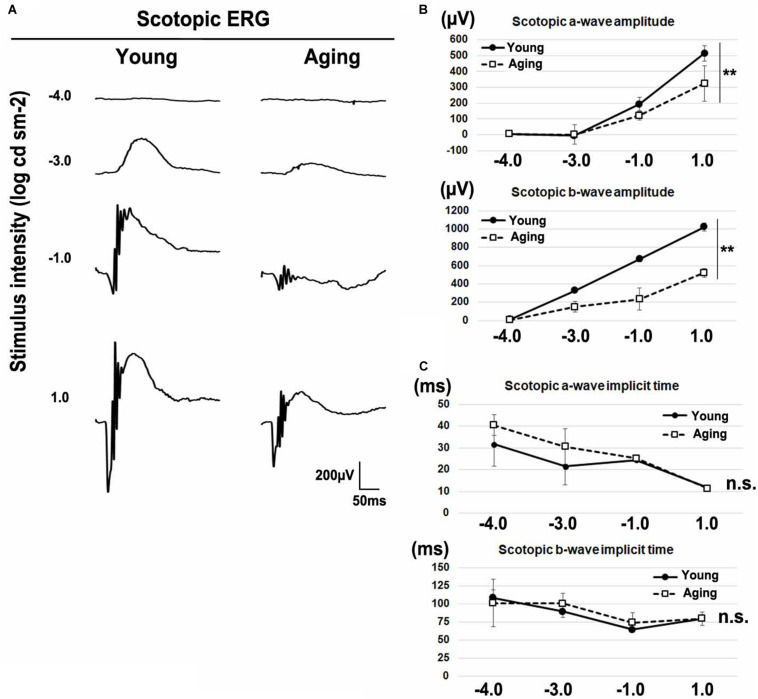
Scotopic ERGs in the aging mice. **(A)** Representative dark-adapted (scotopic) ERGs in the young adult and aging mice induced by four different stimulus intensities (–4.0, –3.0, –1.0, and 1.0 log cd s/m^2^). **(B)** The amplitudes of scotopic a- and b-waves are presented as a function of the stimulus intensity. **(C)** The implicit times of scotopic a- and b-waves are presented as a function of the stimulus intensity. Error bars, mean ± SD (*n* = 3 mice). Two-way repeated measures ANOVA (**p < 0.01, n.s., not significant).

**FIGURE 3:**
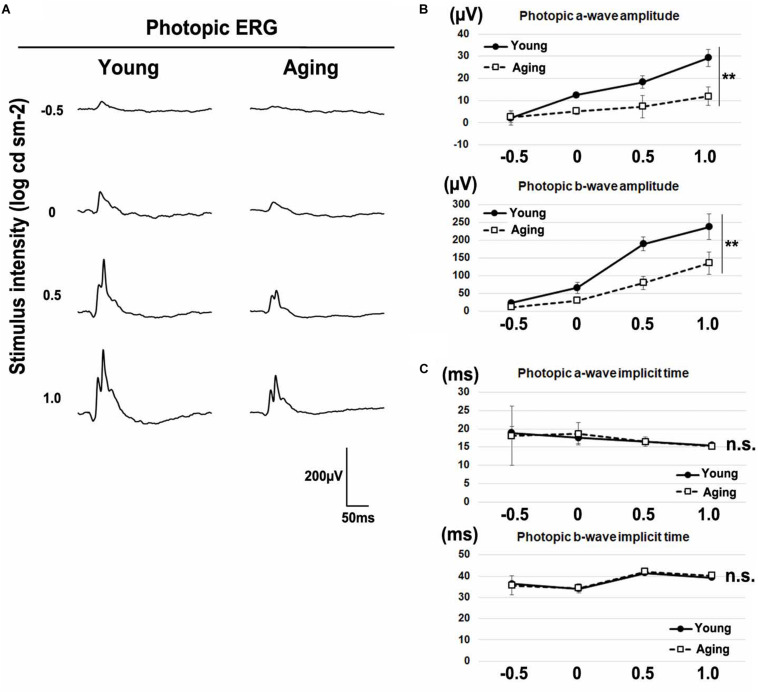
Photopic ERGs in the aging mice. **(A)** Representative light-adapted (photopic) ERGs in the young adult and aging mice induced by four different stimulus intensities (–0.5, 0, 0.5, and 1.0 log cd s/m^2^). **(B)** The amplitudes of photopic a- and b-waves are presented as a function of the stimulus intensity. **(C)** The implicit times of photopic a- and b-waves are presented as a function of the stimulus intensity. Error bars, mean ± SD (*n* = 3 mice). Two-way repeated measures ANOVA (**p < 0.01, n.s., not significant).

Age-related electroretinographic changes have been reported in both rod and cone photoreceptor systems (Gresh et al., 2003; Williams and Jacobs, 2007; Kolesnikov et al., 2010). To confirm whether aging affects the physiological function of the retina, we recorded the ERG responses under dark-adapted (scotopic) and light-adapted (photopic) conditions in young adult and aging mice. We analyzed scotopic ERGs elicited by four different stimulus intensities (−4.0, −3.0, −1.0, and 1.0 log cd s/m^2^) of white light. We found that the amplitudes of a-waves and b-waves significantly decreased in aging mice (Figures 2A,B). The implicit times of scotopic a-waves and b-waves exhibited no significant change between the aging mice and young adult mice (Figure 2C). Next, we recorded photopic ERGs elicited by four different stimulus intensities (−0.5, 0, 0.5, and 1.0 log cd s/m^2^) of white light using young adult and aging mice. In aging mice, the amplitudes of photopic a-waves and b-waves were significantly decreased compared with young adult mice (Figures 3A,B). The implicit times of photopic a-waves and b-waves exhibited no significant change between the aging mice and young adult mice (Figure 3C).

**FIGURE 4:**
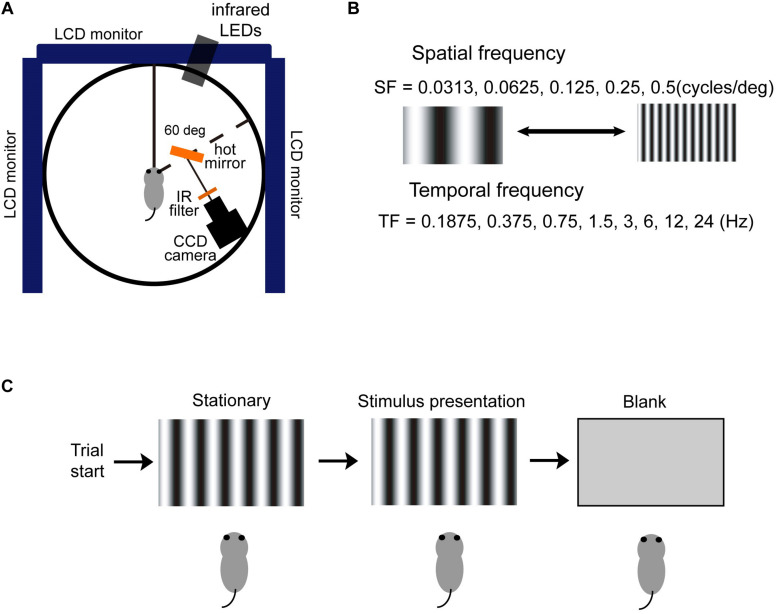
A schematic diagram of the stimuli used for OKR recordings. **(A)** The right eye of each mouse was illuminated by infrared (IR) LEDs and monitored using a CCD camera. Data were analyzed on a computer using an image processing software (Geteye). **(B)** The SFs of the visual stimulus were 0.0313, 0.0625, 0.125, 0.25, and 0.5 cycles/deg. The motion of the visual stimulus was defined by the TF, which was 0.1875, 0.375, 0.75, 1.5, 3, 6, 12, or 24 Hz. **(C)** Scheme of the experimental procedure. A stationary visual pattern was presented and then moved T-N direction (temporal–nasal motion for the right eye) or N-T direction (nasal–temporal motion for the right eye) at a constant speed. After a defined period, the pattern was removed.

### Initial OKRs in the Young Adult and Normal Aging Mice

To characterize the initial OKRs in aging mice, we used visual motion stimuli with a broad range of spatiotemporal frequencies (Figures 4A–C). To study the relationship between eye movements and the spatiotemporal frequency of visual stimuli, we systematically altered the SF of the sine wave and the speed of the visual stimulus in each trial. We first measured and analyzed the initial OKRs in young adult and aging mice. The duration of the visual stimulus motion was 500 ms.

**FIGURE 5:**
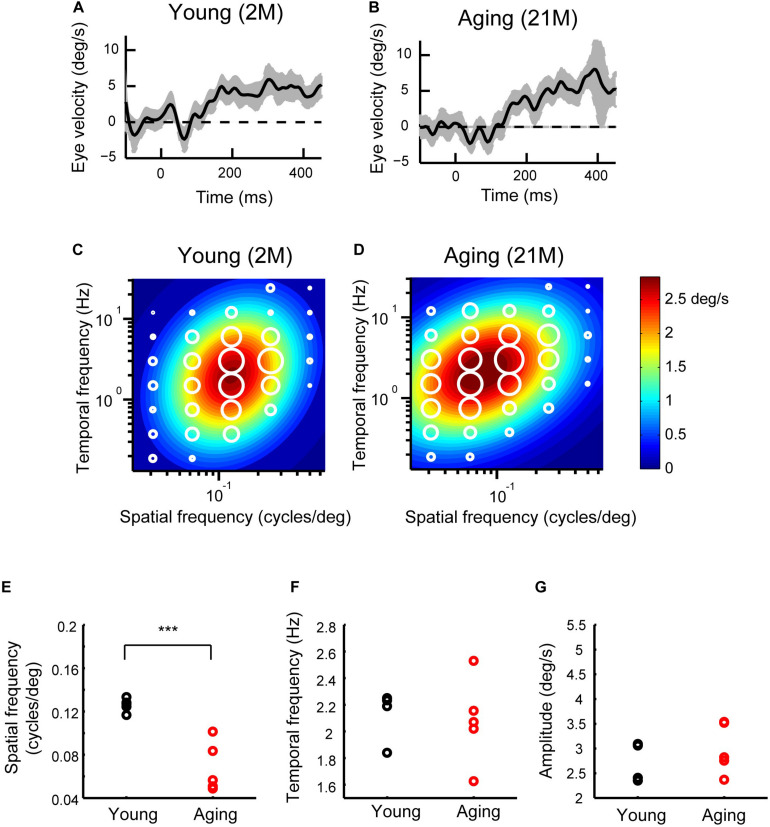
Initial OKRs in the young adult and normal aging mice. **(A,B)** Eye velocity profiles obtained from a young adult mouse **(A)** and an aging mouse **(B)**. The eye movements evoked by the visual stimuli of SF = 0.125 cycles/deg; TF = 1.5 Hz. The gray shaded areas indicate the standard deviation of the individual eye velocity (**A:** 23 trials, **B:** 27 trials). **(C,D)** Heat maps of the initial OKRs in the young adult mice (2–3 months old, *n* = 5) **(C)** and normal aging mice (21–24 months old, *n* = 5) **(D)**. Amplitudes of the initial OKRs represented by diameter of the white circles are plotted in the coordinate system of SF and TF. Heat maps indicate the best-fit Gaussian functions. **(E–G)** Comparisons of the properties of the initial OKRs. The differences in the optimal SF **(E)**, optimal TF **(F)**, and peak amplitudes of the responses **(G)** are shown individual plot (black dots: young adult, red dots: aging). ***p = 2.2 × 10^– 3^, Wilcoxon rank-sum test.

Figures 5A,B show the sample velocity profiles (T-N-N-T profiles) for young adult and aging mice. The OKRs of both young adult and aging mice observed were clear responses (SF and TF were 0.125 cycles/deg and 1.5 Hz, respectively). We calculated the mean eye velocity for each condition, which is presented as response fields. The amplitude of the ocular response is indicated by the diameter of the symbol (Figures 5C,D). The visual responses were largest at a SF of 0.125 cycles/deg and a TF of 3 Hz in both young and aging mice. The responses were reduced as the spatiotemporal frequency deviated from this frequency. In order to further characterize this tuning, 2D Gaussian functions were fitted for each type of mouse (Figures 5C,D). The estimated peaks for the initial OKRs (*sfo*, *tfo*) were 2.93 deg/s at 0.124 cycles/deg, 1.98 Hz for young adult mice (*R*^2^ = 0.86), and 3.02 deg/s at 0.077 cycles/deg, 2.06 Hz for aging mice (*R*^2^ = 0.87). The range of the spatiotemporal frequency that elicited OKRs was similar in the aging and young adult mice, but there was a slight tendency for the optimal SF to be lower in the aging mice compared with young adult mice. To evaluate the significance of these differences, we estimated the optimal spatiotemporal frequencies in individual mice. The data for the individual mice could be characterized using 2D Gaussian functions. All of the young and aging mice showed a successful fit with the 2D Gaussian functions (young: *R*^2^ > 0.61, aging: *R*^2^ > 0.64). The optimal SF was found to be significantly reduced in the aging mice (0.073 ± 0.023 cycle/deg) compared with young adult mice (0.13 ± 0.014 cycles/deg) (Wilcoxon rank-sum test, *p* = 7.9 × 10^–3^) (Figure 5E). The optimal TF was not significantly different between the young adult (1.80 ± 0.73 Hz) and aging mice (2.04 ± 0.30 Hz) (Wilcoxon rank-sum test, *p* = 6.9 × 10^–1^) (Figure 5F). The peak amplitude of the response was not significantly different between the young adult and aging mice (young adult mice, 2.76 ± 0.35 des/s; aging mice, 3.00 ± 0.44 deg/s) (Wilcoxon rank-sum test, *p* = 5.8 × 10^–1^) (Figure 5G). These results indicate that the aging mice exhibit significantly reduced optimal SF but normal TF in the initial OKRs.

### Late OKRs in the Normal Aging Mice

**FIGURE 6:**
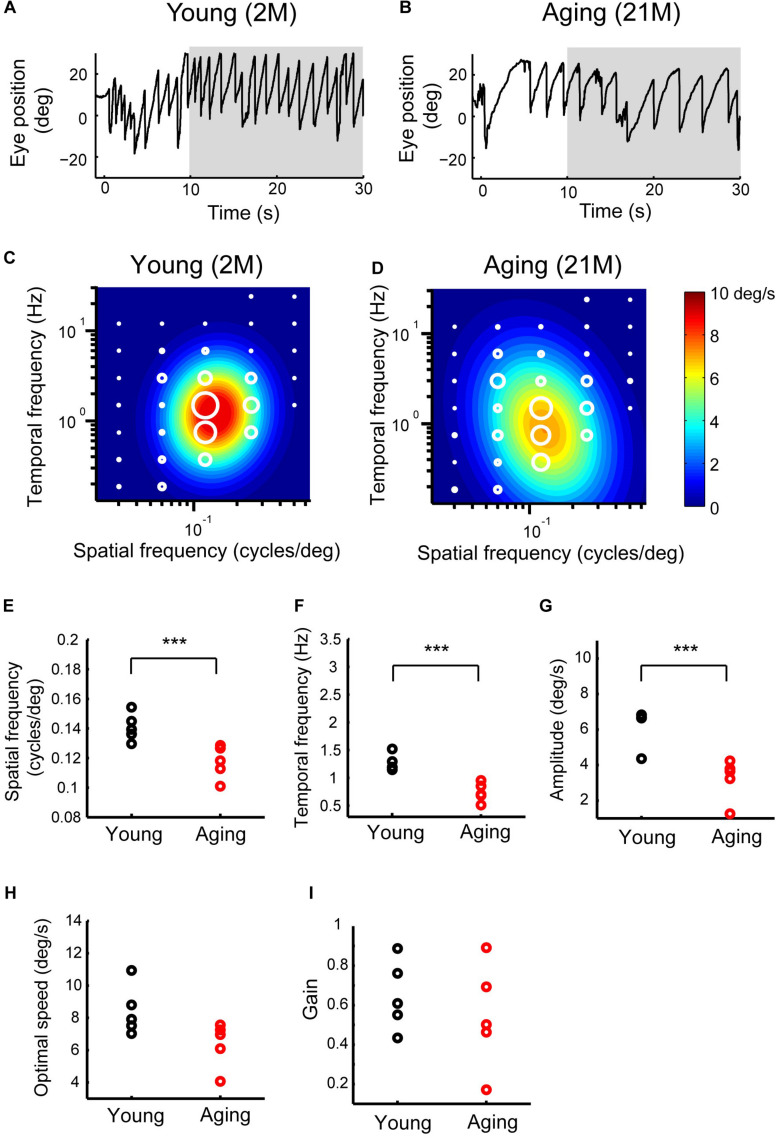
Late OKRs in the young adult and normal aging mice. **(A,B)** Eye position profiles during OKNs in a young adult mouse **(A)** and in an aging mouse **(B)**. Mice were exposed against a moving sinusoidal grating patterns (T-N direction spatial frequency 0.125 cycles/deg, temporal frequency 1.5 Hz, contrast 64%) for 30 s. Slow phase eye velocities during 20-s intervals starting 10 s after the onset of visual motion averaged for each stimulus condition (gray shaded area). **(C,D)** Heat maps of the late OKRs in the young adult mice (2–3 months old, *n* = 5) **(C)** and normal aging mice (21–24 months old, *n* = 5) **(D)**. Mean amplitudes of the slow-phase eye velocity of the late OKRs represented by diameter of the white circles are plotted in the coordinate system of SF and TF. Heat maps indicate the best-fit Gaussian functions **(C,D)**. Comparisons of the properties of the late-phase OKRs. The differences in the optimal SF **(E)**, optimal TF **(F)**, peak amplitudes of the responses **(G)**, stimulus speed at the optimal spatiotemporal frequency **(H)**, and gain at optimal stimuli **(I)** are shown in individual plots (black dots: young adult, red dots: aging). ***p = 7.9 × 10^– 3^, Wilcoxon rank-sum test.

Prolonged exposure to moving sinusoidal grating induces OKN, which consists of slow and quick movements. Figures 6A,B show the eye-position profiles during OKNs in young adult and aging mice. OKNs consisting of a sequence of slow and quick eye movement responses were detected in both young adult and aging mice. To measure the slow-phase eye movements of the OKN, in which the components of quick resetting eye movements are removed from OKN, in young adult, and aging mice, the mean eye velocity was calculated for each experimental condition. The optimal spatiotemporal frequency of young adult mice as estimated from the best-fit 2D Gaussian function (*R*^2^ = 0.924) was 0.161 cycles/deg and 1.56 Hz for SF and TF, respectively (Figure 6C). The aging mice exhibited an optimal SF of 0.126 cycles/deg and an optimal TF of 1.002 Hz (*R*^2^ = 0.927) (Figure 6D). The data for the individual mice could be characterized using 2D Gaussian functions. All of the young and aging mice showed a successful fit with the 2D Gaussian functions (young: *R*^2^ > 0.57, aging: *R*^2^ > 0.56). The optimal SF significantly decreased in aging mice (0.12 ± 0.01 cycles/deg) compared with that in young adult mice (0.14 ± 0.009 cycles/deg) (Wilcoxon rank-sum test, *p* = 7.9 × 10^–3^) (Figure 6E). There was also a significant reduction in the optimal TF in the aging mice (young adult mice, 1.27 ± 0.14 Hz; aging mice, 0.74 ± 0.17 Hz) (Wilcoxon rank-sum test, *p* = 7.9 × 10^–3^) (Figure 6F). The maximal attainable amplitude of the slow-phase velocity was strongly reduced in the aging mice (3.22 ± 1.13 deg/s) compared with young adult mice (5.77 ± 1.27 deg/s) (Wilcoxon rank-sum test, *p* = 7.9 × 10^–3^) (Figure 6G). The speed of the optimal stimulus (calculated by optimal SF/optimal TF) was not significantly changed (young adult mice, 9.06 ± 1.35 deg/s; aging mice, 6.37 ± 1.37 deg/s) (Wilcoxon rank-sum test, *p* = 3.2 × 10^–2^) (Figure 6H). Finally, the gain of the optimal stimulus was not significantly altered (young adult mice, 0.64 ± 0.17; aging mice, 0.54 ± 0.26; Wilcoxon rank-sum test, *p* = 6.9 × 10^–1^) (Figure 6I). These results indicate that the optimal spatiotemporal frequency of the slow-phase of OKN is lower and the amplitude reduced in the normal aging mice compared with young adult mice.

### OKRs in the *4.1G^−/−^* Mice

**FIGURE 7:**
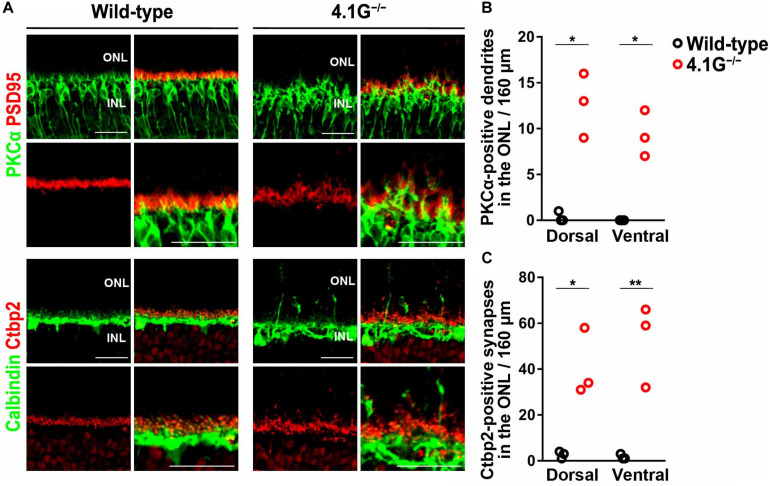
Mislocalization of photoreceptor synaptic terminals observed in *4.1G*^− /−^ retinas. **(A)** Retinal sections of the wild-type control and *4.1G*^− /−^ mice aged 2 months old were immunostained using anti-PSD95, anti-Ctbp2, anti-Calbindin, and anti-PKCα antibodies. **(B,C)** Extended rod bipolar cell dendrites and ectopic photoreceptor terminals were counted. Student *t*-test (*p < 0.05, **p < 0.01). Error bars, mean ± SD (*n* = 3 mice). ONL, outer nuclear layer; OPL, outer plexiform layer; INL, inner nuclear layer. Scale bars, 25 μm.

**FIGURE 8:**
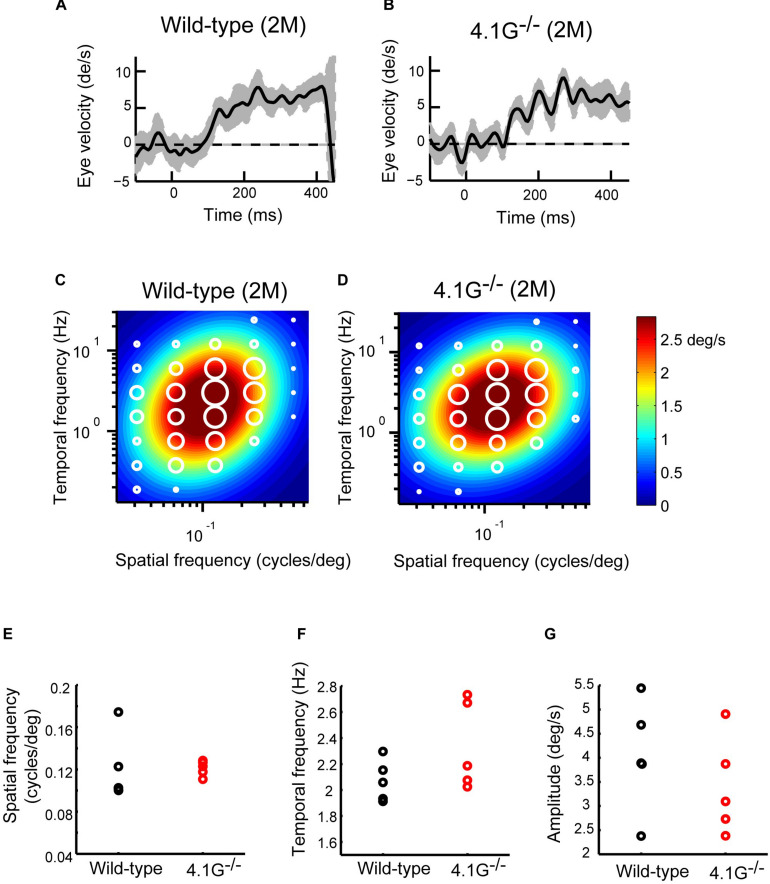
Initial OKRs in young adult *4.1G*^–^*^/^*^–^ mice. **(A,B)** Eye velocity profiles obtained from wild-type control mouse **(A)** and *4.1G*^– /–^ mouse **(B)**. The eye movements evoked by the visual stimuli of SF = 0.125 cycles/deg; TF = 1.5 Hz. The gray shaded areas show the standard deviation of the individual eye velocity (A: 24 trials, B: 18 trials). **(C)** Heat maps of the initial OKRs in young adult wild-type control mice (2–3 months old, *n* = 5) and **(D)** young adult *4.1G*^–^*^/^*^–^ mice (2–3 months old, *n* = 5). Amplitudes of the initial OKRs represented by the diameter of the white circles are plotted in the coordinate system of SF and TF. Heat maps show the best-fit Gaussian functions. **(E–G)** Comparisons of the properties of the initial OKRs. The difference in the optimal SF **(E)**, optimal TF **(F)**, and peak amplitudes of the responses **(G)** are shown in individual plots (black dots: wild-type, red dots: *4.1G*^– /–^).

**FIGURE 9:**
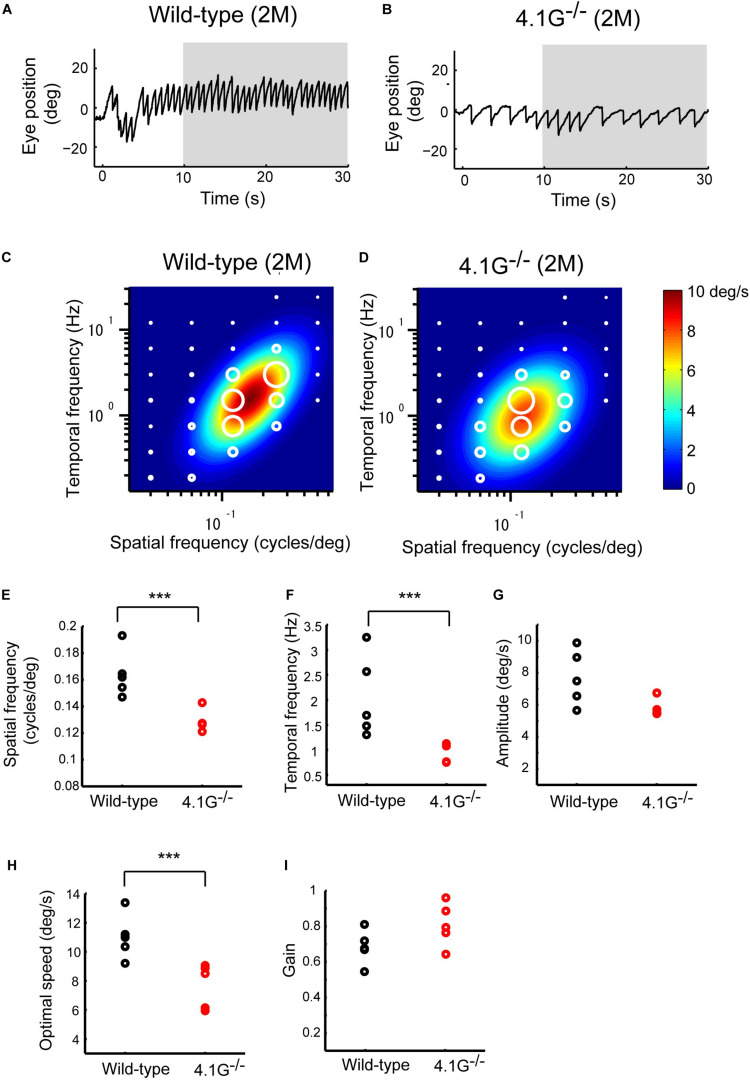
Late OKRs in the young adult *4.1G*^–^*^/^*^–^ mice. **(A,B)** Eye position profiles during OKNs in wild-type control mouse **(A)** and in *4.1G*^– /–^ mouse. **(B)**. Mice were exposed against a moving sinusoidal grating patterns (T-N direction spatial frequency 0.125 cycles/deg, temporal frequency 1.5 Hz, contrast 64%) for 30 s. Slow phase eye velocities during 20-s intervals starting 10 s after the onset of visual motion averaged for each stimulus condition (gray shaded area). **(C)** Heat maps of the late-phase OKRs in young adult wild-type control mice (2–3 months old, *n* = 5) and **(D)** young adult *4.1G*^– /–^ mice (2–3 months old, *n* = 5). Mean amplitudes of the slow-phase eye velocity of the late OKRs represented by diameter of the white circles are plotted in the coordinate system of SF and TF. Heat maps indicate the best-fit Gaussian functions **(C,D)**. Comparisons of the properties of the late-phase OKRs. The differences in the optimal SF **(E)**, optimal TF **(F)**, peak amplitude of the responses **(G)**, stimulus speed at the optimal spatiotemporal frequency **(H)**, and gain at optimal stimuli **(I)** are shown individual plots (black dots: wild-type, red dots: *4.1G*^– /–^). ***p = 7.9 × 10^– 3^, Wilcoxon rank-sum test.

In our previous study, Sanuki et al. (2015) reported that a membrane scaffold protein 4.1G is strongly expressed in photoreceptors in the retina and essential for the correct localization of photoreceptor synapses. *4.1G*^–/–^ mice exhibited impairments of visual acuity in OKN, suggesting the importance of this correct localization of photoreceptor synaptic terminals in the retina for visual function (Sanuki et al., 2015). In the present study, we first immunostained wild-type control and *4.1G*^–/–^ retinas in 2 months old mice using antibodies against rod bipolar cell (PKCα), horizontal cell (Calbindin), and photoreceptor synapse (PSD95 and Ctbp2) markers. The *4.1G*^–/–^ retina showed significantly more extended rod bipolar cell dendrites and ectopic photoreceptor terminals than in the control retina (Figures 7A–C). We measured detailed OKRs for young adult *4.1G*^–/–^ mice using the same visual stimuli as for aging mice. The eye velocity was calculated based on the measured eye position. Sample velocity profiles of the eye movement with a SF stimulus of 0.125 cycles/deg and TF of 1.5 Hz (Figures 8A,B). The data for the individual mice could be characterized by 2D Gaussian functions. All of the young and aging mice showed a successful fit with the 2D Gaussian functions (wild-type: *R*^2^ > 0.51, 4.1G^–/–^: *R*^2^ > 0.66). Although the spatiotemporal tuning and amplitude of the initial OKRs were not significantly different between *4.1G*^–/–^ and wild-type control mice (Figures 8C–G). As shown in Figures 9A,B, OKNs (sequence of slow and fast eye movement responses) were observed in wild-type and *4.1G*^–/–^ mice (SF: 0.125 cycles/deg, TF: 1.5 Hz). To quantify the dependence of late-phase OKRs on spatiotemporal frequency of the visual stimulus, the mean eye velocity was calculated in each experimental condition. The data for control mice can be characterized by the 2D Gaussian function presented in Figure 9C (optimal SF, 0.161 cycles/deg; optimal TF, 1.87 Hz; *R*^2^ = 0.92). The optimal spatiotemporal frequency of *4.1G*^–/–^ mice estimated from the best-fit 2D Gaussian function (*R*^2^ = 0.93) was 0.126 cycles/deg and 1.00 Hz for SF and TF, respectively (Figure 9D). The data for the individual mice could be characterized by 2D Gaussian functions. All of the young and aging mice showed a successful fit with the 2D Gaussian functions (wild-type: *R*^2^ > 0.55, *4.1G*^–/–^: *R*^2^ > 0.53). The optimal SF was significantly lower in *4.1G*^–/–^ mice (0.129 ± 0.007 cycles/deg) than in control mice (0.164 ± 0.017 cycles/deg) (Wilcoxon rank-sum test, *p* = 7.9 × 10^–3^) (Figure 9E). The optimal TF was also significantly lower in *4.1G*^–/–^ mice (0.96 ± 0.18 Hz) than in control mice (2.06 ± 0.79 Hz) (Wilcoxon rank-sum test, *p* = 7.9 × 10^–3^) (Figure 9F). The peak amplitude of the responses was not significantly affected in *4.1G*^–/–^ mice (*4.1G*^–/–^ mice, 6.04 ± 0.63 deg/s; wild-type mice, 7.71 ± 1.65 deg/s) (Wilcoxon rank-sum test, *p* = 9.5 × 10^–2^) (Figure 9G). The speed of the optimal stimulus was significantly reduced in *4.1G*^–/–^mice compared with control mice (*4.1G*^–/–^ mice, 7.69 ± 1.46 deg/s; wild-type mice, 11.0 ± 1.47 deg/s) (Wilcoxon rank-sum test, *p* = 7.9 × 10^–3^) (Figure 9H). The gain of the optimal stimulus was not significantly affected in *4.1G*^–/–^ mice (*4.1G*^–/–^ mice, 0.80 ± 0.11; wild-type mice, 0.70 ± 0.14) (Wilcoxon rank-sum test, *p* = 2.2 × 10^–1^) (Figure 9I).

## Discussion

In the present study, we examined the visual processing of the normal aging and *4.1G*^–/–^ mice by measuring the initial and late phases of OKRs. Both the normal aging and *4.1G*^–/–^mice, which exhibit mislocalization of photoreceptor terminals, showed similar characteristics in their OKRs. We reported two major observations: (1) the optimal SF of the normal aging mice in the initial OKRs was lower than in young adult mice, and (2) the late-phase OKRs in both the normal aging and *4.1G*^–/–^mice exhibited abnormal spatiotemporal characteristics compared with those in young adult mice.

### Photoreceptor Synapse Localization in Visual Function

In the aging mouse retina, neither the total number of cells per retina nor the total numbers or densities of rods and cones were decreased (Trachimowicz et al., 1981; Li et al., 2001; Williams and Jacobs, 2007; Samuel et al., 2011); however, the voltages of both scotopic and photopic ERGs were decreased (Li et al., 2001; Williams and Jacobs, 2007). These previous studies suggest that the abnormalities observed in the ERGs are not due to the decreased numbers of photoreceptors, but possibly due to the reduced synaptic transmission from photoreceptors to the bipolar cells caused by the mislocalization of photoreceptor synaptic terminals. In the current study, we confirmed that scotopic and photopic ERG amplitudes were significantly reduced in the normal aging mice compared with young adult mice (Figures 2A,B, 3A,B). We also confirmed that implicit times in scotopic and photopic ERGs were unaffected in the aging mice compared with young adult mice, as was previously reported (Figures 2C,3C).

The present study demonstrates that optimal SF and TF in the late phase of OKR were significantly reduced in aging mice. We also found that optimal SF in the initial phase of OKR was significantly lower in aging mice compared with young adult mice. However, the mechanisms underlying these abnormalities are unknown. One possibility may be a decrease in the size of retinal ganglion cell (RGC) axon arbors, although the number of RGCs is maintained in the aging retina (Samuel et al., 2011). A second possibility is a change in the receptive field structures involved in the visual processing in the aging retina. Center-surround antagonism is an important receptive field characteristic involved in the SF and TF tuning (Betts et al., 2009; Suematsu et al., 2013; Ho et al., 2018). In the aging retina, center-surround antagonism of photoreceptor and bipolar cells, which are modulated by inhibitory signals from horizontal cells (Baylor et al., 1971; Smith and Sterling, 1990; Dacey et al., 2000; Fahey and Burkhardt, 2003; Zhang and Wu, 2009), might be affected by the mislocalization of photoreceptor synapses, resulting in decreased optimal SF and TF in OKRs.

Samuel et al. (2011) showed that the number of puncta decreased in ON and OFF IPL sublamina layer in aging mice retina. The dendritic arbors of J-RGCs (OFF cells) and BD-RGCs (ON-OFF cells) in aging mice qualitatively resembled those in young adult mice and the axonal arbors of J- and BD- RGCs in the superior colliculus atrophy in aging mice, suggesting that the relay of visual information to central targets may weaken over time. The reduction of axonal density and the terminal area may be associated with a reduced ability of RGCs to convey visual information to the brain (Samuel et al., 2011). These observations imply that aging mice may maintain directional selective ability but undergo reduced RGCs axons and terminals. The initial OKR abnormalities observed in aging retina in the current study may be caused by a reduction of J- and/or BD-RGC axons and terminals. However, to our best knowledge, there are no previous reports on changes of ON RGCs in aging retina. Future studies are needed to determine whether ON RGCs are affected in aging retina, which may underlie the decreased optimal SF and TF in late OKR observed in aging mice.

### OKRs in Comparison With the Previous Studies

Retinal ON and ON-OFF DSGCs contribute to initial and late phases of OKRs (Oyster et al., 1972; Sugita et al., 2013). In particular, ON DSGCs are the main contributors to late phase OKR. Simpson (1984) reported that retinal input to the accessory optic system (AOS) is largely, if not exclusively, from ON DSGCs in rabbit.

In the present study, we found that abnormalities observed in late phase OKRs were more severe than those of initial OKRs in aging mice, indicating that ON DSGCs exert a stronger effect compared to ON-OFF DSGCs in aging. However, it should be noted that the number of ON RGCs and directional selectivity in OKRs are maintained in aging mice. The elucidation of the precise mechanisms on how ON and ON-OFF DSGCs are histologically and functionally affected in response to aging awaits future study.

van Alphen et al. (2009) examined the slow-phase of OKRs of the aging mice that is induced by rotation of a virtual cylinder of sine gratings (van Alphen et al., 2009). They reported that the OKR gain was reduced at SF = 0.17 or 0.25 cycles/deg (contrast: 75%). The current study demonstrates that the optimal spatiotemporal frequency in the late-phase OKRs of the aging mice is lower than that of young adult mice. The impairment described by van Alphen et al. (2009) is consistent with our current results which show inadequate spatiotemporal frequency tuning in the aging mice. However, there is a quantitative difference in the reduced spatiotemporal frequencies in OKRs between our study and that by van Alphen et al. (2009). Conversely, Stahl et al. (2006) reported that the gain of the horizontal and vertical OKRs in C57BL6J mice induced by stimulus rotation was similar between the young adult (2–8 months) and aging mice (>14 months) (Stahl et al., 2006). One possible explanation of the discrepancy may be the difference in visual stimulations used in the current study and the study by Stahl et al.

### OKRs in the Aging and Young Adult *4.1G^−/−^* Mice

The scaffold protein 4.1G is known to be required for proper photoreceptor synapse localization in the mouse retina (Sanuki et al., 2015). The aging mouse retina exhibits mislocalized photoreceptor synaptic terminals (Eliasieh et al., 2007; Kolesnikov et al., 2010; Samuel et al., 2011). We used aging and young adult *4.1G*^–^*^/^*^–^ mice, as both exhibit mislocalized photoreceptor synaptic terminals, to investigate visual processing. The aging mice exhibited impaired OKN, which were similarly observed in the *4.1G*^–^*^/^*^–^ mice. Conversely, both scotopic and photopic ERGs were reduced in the aging mice, but not in the *4.1G*^–/–^ mice. These results suggest two possibilities. First, reduced ERG may not be associated with ectopic processes and OKR abnormalities, which were commonly observed among aging and *4.1G*^–^*^/^*^–^ mice. The other possibility is that ectopic processes observed in aging and *4.1G*^–^*^/^*^–^ mice have different functional consequences, similarly impairing the OKR yet conferring different effects on the ERG. It should be noted that lens and cornea abnormalities, including cataract, in aging mice may affect OKRs. We examined all of the mouse eyes and confirmed that there was no cloudiness before measuring OKRs, suggesting that retinal abnormalities mainly affected OKRs in aging mice in the present study.

### OKRs in Aging Primates

Sekuler et al. (1980) reported that although younger and older human subjects did not differ in their ability to see targets with fine structure (high SFs), old subjects were determined to be less able to see coarse structure (low SFs) than younger subjects by measuring the visual acuity. Older subjects are also less sensitive to moving targets and exhibit lower perceptual efficiency (Sekuler et al., 1980; Wojciechowski et al., 1995; Snowden and Kavanagh, 2006; Bennett et al., 2007; Andersen, 2012). Betts et al. (2005) reported that aging reduces spatial center-surround antagonism in the visual motion processing in humans (Deng et al., 2017). The visual acuity and cone density of the fovea in aged monkeys were significantly reduced compared with those in young monkeys (Ordy et al., 1980). Our data suggest that the spatiotemporal frequencies in OKN in mice were affected at middle and low rather than high spatiotemporal frequencies (Figures 6A,B), whereas the gain in the late OKRs was not significantly different between the young adult mice and aging mice. Conversely, Valmaggia et al. (2004) reported that the OKN gain in humans decreased later in life. The causes of this difference in OKRs between humans and mice are unclear. It should be noted that there is a significant difference in the neural circuitries responsible for OKRs in primates (foveate) and afoveate animals. In afoveate mammals, OKRs are considered to be dominated by subcortical structures (Kato et al., 1988; Hoffmann and Distler, 1989; Mustari and Fuchs, 1989, 1990), as OKRs in rabbits and rats are not influenced by the lesions of the cerebral cortex (Hobbelen and Collewijn, 1971; Harvey et al., 1997). Contrarily, cortical structures are involved in the OKR system in primates (Fuchs and Mustari, 1993; Kawano, 1999; Takemura et al., 2007). There are also critical differences in tuning spatiotemporal frequencies between primates (Miles et al., 1986; Gellman et al., 1990; Sheliga et al., 2005; Gomi et al., 2006; Miura et al., 2006) and mice (van Alphen et al., 2009; Tabata et al., 2010; Sugita et al., 2012; Miura et al., 2018). The optimal SF of the visual stimuli for primate OKRs resembles that in mice, whereas the best TF is quite different; in mice, the best TF was <3 Hz, which is much smaller than that in primates (>16 Hz). The aging processes of humans and animals frequently differ, and not all results from animal experiments directly correspond to humans.

## Data Availability Statement

The raw data supporting the conclusions of this article will be made available by the authors, without undue reservation.

## Ethics Statement

The animal study was reviewed and approved by the Animal Experimental Committees of the Institute for Protein Research (approval ID 29-01-3) at Osaka University.

## Author Contributions

YS and TF conceived and designed the experiments. HY and YM performed mouse mating, histological, and ERG experiments. YS performed the OKR experiments and analyzed the data. YS, HY, and TF wrote the manuscript. All authors contributed to the article and approved the submitted version.

## Conflict of Interest

The authors declare that the research was conducted in the absence of any commercial or financial relationships that could be construed as a potential conflict of interest.
